# Combining Immune Checkpoint Inhibitors With Conventional Cancer Therapy

**DOI:** 10.3389/fimmu.2018.01739

**Published:** 2018-07-27

**Authors:** Yiyi Yan, Anagha Bangalore Kumar, Heidi Finnes, Svetomir N. Markovic, Sean Park, Roxana S. Dronca, Haidong Dong

**Affiliations:** ^1^Division of Medical Oncology, Mayo Clinic, Rochester, MN, United States; ^2^Department of Immunology, Mayo Clinic, Rochester, MN, United States; ^3^Department of Pharmacy, Mayo Clinic, Rochester, MN, United States; ^4^Department of Radiation Oncology, Mayo Clinic, Rochester, MN, United States; ^5^Division of Hematology and Medical Oncology, Mayo Clinic, Jacksonville, FL, United States; ^6^Department of Urology, Mayo Clinic, Rochester, MN, United States

**Keywords:** immunotherapy, chemotherapy, targeted therapy, radiotherapy, combination therapy

## Abstract

Immune checkpoint inhibitors (ICIs) have recently revolutionized cancer treatment, providing unprecedented clinical benefits. However, primary or acquired therapy resistance can affect up to two-thirds of patients receiving ICIs, underscoring the urgency to elucidate the mechanisms of treatment resistance and to design more effective therapeutic strategies. Conventional cancer treatments, including cytotoxic chemotherapy, radiation therapy, and targeted therapy, have immunomodulatory effects in addition to direct cancer cell-killing activities. Their clinical utilities in combination with ICIs have been explored, aiming to achieve synergetic effects with improved and durable clinical response. Here, we will review the immunomodulatory effects of chemotherapy, targeted therapy, and radiation therapy, in the setting of ICI, and their clinical implications in reshaping modern cancer immunotherapy.

## Introduction

Deeper understanding in the regulatory mechanisms of antitumor immunity, especially the identification of immune checkpoint pathways, has led to the success of modern immunotherapy. The past decade has witnessed a revolution in cancer therapy since the introduction of immune checkpoint inhibitors (ICIs), including anti-CTLA4 antibody and anti-PD-1/PD-L1 antibody. These antibodies have reshaped the landscape of treatments in various types of cancers, including melanoma, renal cell cancer, colorectal cancer (CRC), and non-small-cell lung cancer (NSCLC). However, it is estimated that up to 60–70% of patients do not respond to single-agent ICI therapy (1–7). To address this clinical challenge, different conventional cancer treatment modalities have been tested in combination with ICIs to achieve synergetic effects and to overcome the resistance to immunotherapy. Although some of these approaches have provided clinical benefits, the lack of knowledge in the functional interactions between conventional cancer therapies and immune checkpoint blockades at the molecular level remains a crucial hurdle in developing rational and optimal combination strategies. In this article, we will review the immune-regulatory effects of conventional cancer treatments and their clinical applications in combination with immune checkpoint blockades and future challenges.

## Combination of Immunotherapy and Chemotherapy

### Immunomodulatory Impact of Cytotoxic Chemotherapy

It has long been speculated that the immunoregulatory properties of conventional cytotoxic chemotherapy contribute to the antitumoral effects of these agents, in addition to direct tumor killing (8). Although the mechanisms are yet to be fully understood, chemotherapy can regulate antitumor T cell response through increasing tumor antigenicity, inducing immunogenic cell death (ICD), disrupting immune suppressive pathways, and enhancing effector T-cell response (9–12).

Chemotherapy executes direct cancer killing *via* multiple mechanisms, including causing DNA damage, inhibiting DNA replication, and preventing mitosis (13). The induced tumor cell death further elicits systemic and intratumoral immune responses, contributing to the antitumor immunity. Chemotherapy enhances the antigenicity of the tumors through the increase of mutation burden and neoantigen load (such as in NSCLC and other various malignancies), which are correlated with higher responses to ICI therapy (14, 15). Some chemotherapy drugs upregulate MHC class 1 expression to increase antigen presentation (16, 17). In addition, chemotherapy drugs promote dendritic cell maturation and enhance the T cell activation by DCs (18). Chemotherapy also promotes ICD by releasing damage-associated molecular patterns, which can generate effector immune response when bound to pattern-recognition receptor. Experiments in animal models have suggested that some chemotherapy drugs induce the expression of PD-L1 on ovarian cancer cells (19).

Cytotoxic chemotherapy is regarded as immunosuppressive due to its dose-limiting myelosuppression. However, recent studies have demonstrated that it also can disrupt suppressive pathways. These immunosuppressive subsets play critical roles in downregulating the antitumor T-cell response and in promoting resistance to ICI treatments. Lymphodepletion resulted after chemotherapy can potentiate antigen-specific T-cell responses, therefore, augment antitumor immunity, particularly during the recovery phase from lymphopenia. Lymphodepletion can eliminate regulatory T cells (Treg) and other immunosuppressive cell subsets, such as myeloid-derived suppressor cells (MDSCs) (20–22) and tumor-associated macrophages (TAMs) (23). For instances, cyclophosphamide eliminates Treg and improves overall survival when combined with immunotherapy in a colon cancer models (24). Doxorubicin eliminates MDSCs and enhances the efficacy of immunotherapy in breast cancer (25). Reductions of these immunosuppressive populations in the tumor microenvironment of glioblastoma, synergize with anti-PD-1 therapy, and enhance the antitumor immunity (26, 27). The elimination of these immunosuppressive cells will increase the availability of survival and proliferative cytokines for T cells and lower the threshold for T-cell activation. Chemotherapeutic reagents also promote the polarization of Th1/Th2 and enhance the proliferation of T-lymphocytes in patients with advanced solid cancers (such as renal cell carcinoma, colon cancer, and ovarian cancer) (28, 29). Over the past decade, multiple studies have shown that different types of chemotherapy drugs can modulate the antitumor immunity in various mechanisms (9).

Given the extensive roles of chemotherapy in regulating the antitumor immune response, it is safe to hypothesize that the addition of chemotherapy to ICI may further enhance the activities of cytotoxic T cells with improved clinical outcomes. Over the past few years, chemoimmunotherapy (CIT) combination has attracted attention from clinicians and researchers and has been investigated in multiple clinical trials.

### Clinical Studies With CIT Combinations

Chemotherapy in combination with immunotherapy (CIT) has been studied in multiple solid tumors, largely in NSCLC, providing broadened treatment options with improved outcomes.

The combination of pembrolizumab with pemetrexed and carboplatin has been evaluated in KEYNOTE-021, a multicenter phase 1/2 study, in patients with NSCLC. In the phase 1 study (30), pembrolizumab in combination with either carboplatin and paclitaxel, or carboplatin and paclitaxel plus bevacizumab, or carboplatin and pemetrexed was investigated, with overall response rate (ORR) of 52, 48, and 71%, respectively, irrespective of PD-L1 expression levels. These results led to the phase 2 study, evaluating the clinical outcome of pembrolizumab in combination with carboplatin and pemetrexed (31). A total of 123 chemotherapy-naïve nonsquamous NSCLC patients were randomized to chemotherapy alone, or chemo-pembrolizumab combination. Indefinite pemetrexed maintenance therapy was allowed for patients in chemotherapy alone group, and maintenance therapy with indefinite pemetrexed and up to 24 months of pembrolizumab was allowed for patients in chemo-pembrolizumab combination group. A significantly higher response rate was observed in the CIT combination group (55%) than in the chemotherapy alone group (29%), with progression-free survival (PFS) of 13 vs. 6 months, respectively. The magnitude of adverse effects (grade 3 or above) in both the groups was comparable (39 vs. 26%, respectively). Based on this study, the FDA granted accelerated approval of pembrolizumab in combination with carboplatin and pemetrexed for the treatment of NSCLC adenocarcinoma in the first-line setting. Updated survival data with median follow-up of 18.7 months showed a PFS of 19.0 months in CIT group vs. 8.9 months in chemotherapy group, with OS in CIT group not reached vs. 20.9 months in the chemotherapy arm (32).

In KEYONOTE-021 study, the tumor cell-associated PD-L1 expression level can impact the response rates in patients who received CIT treatment: response rate of 57% in those with <1% PD-L1 expression, 54% in those with ≥1% PD-L1 expression, 26% in those with 1–49% PD-L1 expression, and 80% in those with ≥50% PD-L1 expression (30). Accordingly, higher cutoff of PD-L1 expression was associated with higher response rates. Since pembrolizumab singe-agent is only indicated in NSCLC patients with ≥50% PD-L1 expression in the frontline setting (33, 34), this study established CIT as an alternative first-line therapeutic approach for nonsquamous NSCLC patients with <50% tumor PD-L1 expression, who do not harbor targetable mutations.

The results of the phase 3 trial (KEYNOTE-189) evaluating pembrolizumab in combination with chemotherapy in frontline setting in nonsquamous NSCLC patients without sensitizing EGFR or ALK mutations have been recently reported. A total of 616 patients were randomized in a 2:1 ratio to receive pemetrexed and platinum-based drug plus either pembrolizumab or placebo for 4 cycles, followed by pembrolizumab or placebo (for up to 35 cycles) plus pemetrexed maintenance therapy. With a median follow-up of 10.5 months, the 12-month OS was 69.2% in combination group vs. 49.4% in placebo group, and median PFS was 8.8 vs. 4.9 months, respectively. Interestingly, the survival benefit was seen across all PD-L1 categories (35).

A phase 3 trial evaluating CIT combination in frontline setting is currently ongoing for squamous NSCLC (KEYNOTE-407). The results from a second interim analysis after patients had been followed for a median of 7.8 months were recently presented (36). A total of 559 patients were enrolled and stratified prior to randomization based on tumor PD-L1 expression (<1 vs. ≥1%) as well as the choice of taxane (paclitaxel vs. nab-paclitaxel). Patients were assigned to receive four cycles of carboplatin and paclitaxel/nab-paclitaxel plus either pembrolizumab or placebo, followed by pembrolizumab (for CIT group) or placebo (for placebo group) maintenance therapy. Patients in the placebo group could cross over to receive pembrolizumab monotherapy after disease progression. Significant improvements in OS, PFS, and response rate were observed in CIT group vs. chemo-alone group regardless of PD-L1 expression level: the median OS of 15.9 vs. 11.3 months (HR 0.64, 95% CI [0.49, 0.85]; *p* = 0.0008), PFS of 6.4 vs. 4.8 months (HR 0.56, 95% CI [0.45, 0.70]; *p* < 0.0001), and objective response rate of 58.4 vs. 35.0% at the first interim analysis (*p* = 0.0004), respectively.

Nivolumab has also been tested in multiple clinical trials. In phase 1 CHECKMATE-012 trail, 56 patients with NSCLC received first-line therapy with combination of nivolumab with either gemcitabine and cisplatin, pemetrexed and cisplatin, or paclitaxel and carboplatin, followed by nivolumab maintenance therapy (37). CIT combination demonstrated improved ORR compared with nivolumab monotherapy in the front-line setting, with a manageable, non-overlapping toxicity profile. While nivolumab monotherapy has shown an ORR of 23% in the historical study (38), it demonstrated a higher ORR when combined with gemcitabine and cisplatin (33%), or pemetrexed and cisplatin (47%), or paclitaxel and carboplatin (43%), with acceptable tolerability and toxicity profiles. In addition to that, association between treatment response and PD-L1 expression levels was not observed (ORR 48% in PD-L1 ≥ 1 vs. 43% in PD-L1 < 1%).

A phase 3 study (CHECKMATE-227) is ongoing to evaluate nivolumab in combination with chemotherapy vs. chemotherapy alone in the frontline setting for patients with NSCLC (39). A total of 550 chemo-naive NSCLC patients without known sensitizing EGFR/ALK mutations, with <1% tumor PD-L1 expression were randomized to receive nivolumab 3 mg/kg Q2W + ipilimumab 1 mg/kg Q6W, nivolumab 360 mg Q3W + chemo, or chemotherapy for 2 years. In the recently presented results (40), a total of 177 patients received CIT while 186 received chemotherapy alone. With a minimum follow-up of 11.2 months, an improved PFS was seen in nivolumab-chemo arm vs. chemotherapy alone arm (HR = 0.74, 95% CI [0.58, 0.94]). Part 2 of CheckMate 227 is currently ongoing to evaluate the benefit of nivolumab-chemo combinational irrespective of PD-L1 expression.

The FDA approval of the pembrolizumab-chemotherapy combination in NSCLC has encouraged physicians to investigate various CIT in clinical trials in other types of cancers (Table 1). The combination of pembrolizumab with different chemotherapy regimens is being evaluated in PembroPlus study (NCT02331251) for patients with various types of advanced cancers. Pembrolizumab in addition to cisplatin or capecitabine or 5-Fluorouracil is being investigated in KEYNOTE-062 study (NCT02494583) for patients with advanced gastric cancer. Atezolizumab, an anti-PD-L1 antibody, has also been evaluated in combination with chemotherapy in the phase 1 GP28328 study (NCT01633970) in multiple tumor types, demonstrating improved ORR of CIT in patients with NSCLC (41). Phase 3 studies are the currently ongoing to determine the clinical benefit of atezolizumab in combination with chemotherapy (IMpower 130, IMpower132). Durvalumab, another anti-PD-L1 antibody, is also being evaluated in a phase 3 clinical trial (POSEIDON), randomizing untreated NSCLC patients to chemotherapy alone or chemotherapy in combination with durvalumab with or without anti-CTLA4 antibody, tremelimumab (NCT03164616).

**Table 1:** Completed and ongoing clinical trials evaluating chemotherapy in combination with immune checkpoint inhibitors.

Trial	Tumor type	Therapy regimes	Endpoints
Gadgeel et al. (30)	NSCLC (KEYNOTE 021) phase 1	Cohort A: Pembrolizumab (pembro) + carboplatin and paclitaxel (CP) → pembro Cohort B: Pembro + CP + bevacizumab (BEV) → pembro + BEV Cohort C: Pembro + carboplatin + pemetrexed (PEM) → pembro + PEM	Overall response rate (ORR): 52% Progression-free survival (PFS): 10 months ORR: 48% PFS: NR ORR: 48% PFS: 10

Langer et al. (31)	NSCLC (KEYNOTE 021) phase 2	Pembro + carboplatin + PEM → pembro + PEM Carboplatin/PEM → PEM	ORR: 55% PFS: 19 months ORR 29% PFS: 8.9 months
Borghaei et al. (32)			

Gandhi et al. (35)	NSCLC (nonsquamous) (KEYNOTE-189)	Pembro + platinum + PEM → pembro + PEM Placebo + platinum + PEM → PEM	OS (12 months): 69.2% PFS: 8.8 months OS (12 months): 49.4% PFS: 4.9 months

KEYNOTE-047 (ongoing)	NSCLC (squamous)	Pembro + CP → Pembro Placebo + CP	Primary: OS and PFS Secondary: ORR

Weiss et al. (42)	Advanced, metastatic solid tumors (PEMBRO-PLUS)	Pembrolizumab plus gemcitabine (G), G + docetaxel (D), G + nab-paclitaxel (NP), G + vinorelbine (V), or irinotecan	Standard dose pembrolizumab can be safely combined with G, G + NP, G + V, I, and LD

Rizvi et al. (37)	NSCLC (CHECKMATE 012) phase 1	Nivolumab (Nivo) + gemcitabine (GEM) + cisplatin (CIS) → Nivo Nivo + PEM the + CIS → Nivo Nivo (10 mg/kg) + CP →Nivo Nivo (5 mg/kg) + CP → Nivo	PFS: 5.7 months OS: 11.6 months PFS: 6.8 months OS: 19.2 months PFS: 4.8 months OS: 14.9 months PFS: 7.1 months OS: NR

Paz-Ares et al. (39)	NSCLC (CHECKMATE 227) phase 3 Ongoing	Chemotherapy alone or in combination with Nivo Squamous: CP Nonsquamous: PEM + carboplatin (or cisplatin)	Primary: PFS and OS Secondary: ORR

Liu et al. (41)	NSCLC	Atezolizumab (Atezo) + CP → Atezo Atezo + carboplatin/PEM → Atezo + PEM Atezo + carboplatin + nab-paclitaxel → Atezo	ORR: 36% PFS: 7.1 months OSS: 12.9 months ORR: 68% PFS: 8.4 months OS: 18.9 months ORR: 46% PFS: 5.7 months OS: 17.0 months

NCT02367781 NCT02367794 NCT02657434	IMpower 130 (NSCLC nonsquamous) IMpower 131 (NSCLC Squamous) IMpower 132 (NSCLC nonsquamous)	Atezo + carboplatin + nab-paclitaxel → Atezo Atezo + CP → Atezo Atezo + PEM/carboplatin (or cisplatin) → Atezo + PEM	Primary: PFS and OS Secondary: ORR
	All ongoing		

NCT02537418	NSCLC (PESEIDON) Phase 3 Ongoing	Durvalumab + tremelimumab + chemotherapy (histology-based) Durvalumab + chemotherapy (histology-based) Chemotherapy (histology-based)	Primary: PFS Secondary: OS and ORR

NCT02735239	Metastatic/locally advanced esophageal cancer (neoadjuvant therapy)	Durvalumab in combination with standard of care chemotherapy or chemoradiation	Primary: Adverse events, dose-limiting toxicities Secondary: ORR, PFS, OS

NCT03317496	NSCLC Urothelial Cancer	Avelumab + pemetrexed/carboplatin Avelumab + gemcitabine/cisplatin	Primary: Confirmed OR Secondary: PFS, OS, duration of response, time to tumor response

Govindan et al. (43)	Advanced NSCLC	Ipilimumab + CP Placebo + CP	OS: 13.5 months PFS: 5.6 months (with higher toxicities) OS: 12.4 months PFS: 5.6 months

Patel et al. (44)	Metastatic melanoma	Ipilimumab plus temozolomide	6-month PFS was 45% with median OS of 24.5 months. 10 (15.6%) confirmed partial responses and 10 (15.6%) confirmed complete responses. No deaths/unexpected toxicities

Reck et al. (45)	SCLC	Ipilimumab + etoposide and platinum Placebo + etoposide and platinum	OS: 11.0 months PFS: 4.6 months OS: 10.9 months PFS: 4.4 months

Yamazaki et al. (46)	Melanoma	Ipilimumab + dacarbazine	Was not considered tolerable in the Japanese patient population

### Considerations and Challenges

Despite recent clinical success, our limited understanding of the interplay between chemotherapy and immunotherapy hurdles the design of the optimal combination strategy. Different types of chemotherapies execute cytotoxicity against tumors through distinct mechanisms. Similarly, each chemotherapy drug demonstrates unique impact on the systemic and intratumoral immune responses (8). Distinct intrinsic signaling pathways in different types of tumors attribute to the variability in their chemosensitivities. Moreover, the advanced knowledge of immune checkpoint pathways has rapidly expanded the list of ICIs that are acting through different mechanisms (e.g., TIM-3, LAG-3, indoleamine 2,3-dioxygenase, B7-H3). Understanding the impact of chemotherapy in the setting of different types of immunotherapies, as well as the impact of immunotherapy on chemosensitivity (or chemoresistance) of tumors, at both cellular and molecular levels are crucial for the design of rational combination regimens with minimized toxicity.

It is not just the appropriate combination but also the sequence and scheduling of CIT that have to be considered in the clinical scenario. In most of the clinical trials, chemotherapy and immunotherapy are given concurrently, lacking the understanding of the impact of sequencing on the antitumor immunity. Given the fact that antitumor T cell response has different phases that can be targeted by different ICIs (47, 48), and that chemotherapy can modulate immune system while having cytotoxicity against T cells at the same time, it is very likely that the sequence and timing of CIT would significantly impact the treatment outcomes. Both preclinical and clinical studies have shown controversial results regarding the sequence of CIT, and the ideal combination regimens are evolving. Since chemotherapy can cause immunogenic tumor death that promotes T cell priming, some have thought that immunotherapy should be given after chemotherapy to allow maximal T cell proliferation and expansion. In a phase 2 study investigating carboplatin and paclitaxel in combination with concurrent or sequenced ipilimumab in small-cell lung cancer (SCLC), the sequenced treatment is associated with improved PFS compared with chemotherapy alone (49). However, in a mesothelioma mouse model, concurrent treatment with ipilimumab and chemotherapy improved outcomes compared with sequential therapy (50). By contrast, studies in lung cancers and melanoma have shown that chemotherapy administered after immunotherapy can achieve successful clinical responses (51, 52). Our group recently demonstrated that in metastatic melanoma patients who had disease progression after anti-PD1 monotherapy, the addition of chemotherapy to PD-1 blockade induced a significantly improved clinical response, with an ORR of 65% (CR of 25%) (53–55). Using peripheral blood from patients who benefited from the CIT combination, we identified a novel subset of therapy-responsive CD8^+^ T cells (CX3CR1^+^) that can survive chemotherapy toxicity with preserved CTL functions (53). This subset of effector T cells is less actively proliferating during the combination therapy, and hence is spared from chemocytotoxicity. In addition, these CX3CR1^+^CD8^+^ T cells demonstrate the ability to efflux chemotherapy drugs. Our preclinical animal model studies also demonstrate that that CIT combination after previous exposure to immunotherapy provides better tumor control with an increase in CX3CR1^+^CD8^+^ T cells population (53). Our results elucidated mechanisms that are responsible for the success of combination, facilitating the rational design of CIT. This subset of T cells may be used as a biomarker in monitoring and predicting clinical response to CIT, especially when tumor PD-L1 levels fail to show direct correlation with the CIT treatment outcomes in multiple clinical trials. Future studies are warranted to define whether the efficacy of CIT is dependent on certain ICI to recruit immune cells into tumor tissues (like CX3CR1^+^CD8 effector T cells) or to expand local tumor-infiltrating immune cells to reject tumors.

The scheduling and timing of chemotherapy in CIT are also critical for achieving clinical success. The fluctuation of dynamic systemic immunity in metastatic melanoma patients has been reported. Chemotherapy that was delivered in synchronization with unique phase of dynamic immune response tends to correlate with improved response (56). Some chemotherapy drugs work in cell cycle specific manners (e.g., S phase for drugs inhibiting DNA synthesis), suggesting that their direct tumor-killing activities and immunomodulatory effects can be influenced by the schedule of drug administrations. Platinum-based chemotherapy given in different dosing schedules has shown different antitumor immune responses associated with variable clinical outcomes in an ovarian cancer mouse model (57). Our recent preclinical study further demonstrated that the timing of chemotherapy administration after the immunotherapy initiation can affect the frequencies of CX3CR1^+^ T cell population and the treatment outcomes (53), suggesting the variable chemo-induced immunomodulation in relationship to the timing of the immunotherapy. With the overwhelming possibility of CIT combinations, further preclinical and clinical research is in need to design rational combinations for different types of cancers, while minimizing the therapeutic toxicities.

## Combination of Immunotherapy and Molecular Targeted Therapy

The identification of deregulated cellular signaling pathways that are responsible for tumorigenesis has led to the successful development of molecular targeted therapy in recent decades. Medications inhibiting oncogenic pathways, DNA repair response, and angiogenesis pathways have provided effective treatment options for patients with different types of malignancies, although response durability is often lacking. Recent research has demonstrated that these pathways also have immunomodulatory effects on systemic and intratumoral antitumor immune responses, suggesting that the combination of molecular targeted therapy with ICIs can result in synergistic antitumor effects.

### BRAF and MEK Inhibitors

Dysregulations in the RAS/RAF/MAPK pathway are commonly seen in oncogenic transformation and tumorigenesis. Mutations in *BRAF*, a proto-oncogene, are associated with various types of cancers, especially melanoma. In patients with BRAF mutant metastatic melanoma, high response rates are observed after treatment with BRAF/MEK inhibitors, although the duration of response is short lasting due to adaptive therapy resistance. Since ICIs provide durable clinical benefit, combinations of ICIs with BRAF/MEK inhibitors may provide fast and long-lasting disease control.

In melanoma cell models, BRAFV600 mutations can lead to decreased antitumor immunity through upregulation of immunosuppressive factors [e.g., IL-10, vascular endothelial growth factor (VEGF)] (58), elevation of PD-L1 expression levels (59), increased tumor infiltration of immunosuppressive cells (e.g., Treg) (60), and downregulation of melanoma MHC-1 expression (61). In patients with metastatic melanoma, BRAF inhibitors have shown to decrease the immunosuppressive cytokines with resultant increased CD8^+^ T cell tumor infiltration and antitumor immunity (62). In addition, the treatment response of BRAF inhibitors is CD8^+^ T-cell dependent (60, 63). Interestingly, in CRC patients, the majority of those who have PD-L1 positive tumor carry BRAF mutations along with microsatellite instability, suggesting that the immunosuppressive tumor microenvironment can be induced by BRAF mutations (64). In a colorectal carcinoma mouse model, MEK inhibitors demonstrated synergistic therapeutic effects with anti-PD-L1 antibody (65). In a melanoma mouse model, dabrafenib treatment alone results in increased TAMs and Treg, while the addition of trametinib further decreases these suppressive cell subsets. When combined with anti-PD-L1 antibody, the triple therapy provides a superior tumor control (66).

The combination of BRAF inhibitors with anti-CTLA-4 antibody has been studied in multiple clinical trials. However, substantial immune-related adverse effects were the main concern in several studies. Liver toxicity and high-grade skin adverse effects were seen in trials with vemurafenib and ipilimumab combination (NCT01400451 and NCT01673854) (67, 68). Severe colitis was seen in patients who received dabrafenib, trametinib, and ipilimumab triple combination (NCT01767454). Anti-PD1/PD-L1 antibodies were also evaluated in combination with BRAF/MEK inhibitors (Table 2). In a phase 1 study, the combination of dabrafenib, trametinib, and durvalumab demonstrated tolerable toxicity profiles and encouraging disease response rates (69). Pembrolizumab is also being studied in combination with dabrafenib and trametinib in patients with metastatic melanoma (NCT02130466). Multiple other clinical trials are currently ongoing (NCT01940809; NCT01656642; NCT02027961; NCT02224781) to test similar combinations.

**Table 2:** Clinical trials of BRAF targeted therapy in combination with immune checkpoint inhibitors.

Trial	Mutation status	Therapy regimens	Outcomes
Puzanov et al. (70)	BRAF V600 mutant melanoma	Dabrafenib ± trametinib + ipilimumab	Triple therapy resulted in severe GI toxicities
Ribas et al. (67)	BRAF V600 mutant melanoma	Vemurafenib + ipilimumab	Combination resulted in severe liver toxicities
Ribas et al. (69)	Both wild-type and BRAF mutant melanoma	Durvalumab + dabrafenib + trametinib Durvalumab + trametinib Trametinib → durvalumab	Tolerable, no unexpected toxicity
Amin et al. (68)	BRAF V600 mutant melanoma	Vemurafenib + ipilimumab	Combination resulted in high-grade GI and skin toxicities
NCT02224781	Metastatic melanoma	Dabrafenib + trametinib followed by ipilimumab + nivolumab at progression vs. ipilimumab + nivolumab followed by dabrafenib + trametinib	Ongoing Primary: OS Secondary: PFS

Combining BRAF/MEK inhibitors with ICIs has the potential to overcome resistance to targeted therapy; however, further investigations are needed to understand the underlying molecular interplay and to design the ideal combination regimens. Given the toxicities observed in early trials, the optimum tolerable dose of targeted therapy in combination with ICIs needs to be determined. The rapid development of resistance to BRAF/MEK inhibitors and their dynamic impacts on the tumor microenvironment and systemic antitumor immunity should also be considered to determine the sequencing and scheduling of the combination. One study demonstrated long-term tumor control after short-term targeted therapy with subsequent anti-PD1 antibody in patients with metastatic melanoma, and T cell tumor infiltration was seen in tumor biopsies within 1 week after BRAF/MEK inhibitors administration while less frequent after 2 weeks on therapy (71), suggesting that the timing of combination with PD-1 blockade can impact patient outcomes.

Other oncogenic pathways, such as PI3K–Akt–mTOR pathway and KIT, can also regulate the antitumor immunity in addition to regulating cellular proliferation, providing further options for combination therapy with ICIs. For example, inhibition of PI3kγ can promote T cell infiltration through regulating the balance between stimulatory and suppressive TAMs (72). Inhibition of KIT decreased the INF-γ induced PD-L1 expression (73). Treatments targeting these pathways are also being investigated in combination with ICIs to overcome their limited clinical response. The crosstalk between multiple signaling oncogenic pathways in the setting of immunotherapy should be further investigated to determine the ideal drugs to be combined, with special consideration of the individual’s unique intrinsic genetic background.

### Poly (ADP-Ribose) Polymerase (PARP) Inhibitors

DNA damage repair machinery plays important roles in cell cycle regulation and tumorigenesis (74). Inhibition of DNA damage repair can potentially increase the tumor mutational burden, especially in tumors with high endogenous DNA damage. PARP plays a critical role in the repair of single-strand DNA break. In tumor cells with BRCA mutations, PARP inhibition can increase the genomic instability and cell death, with resultant increased neoantigen load and antitumor T cell response (75). This synthetic lethality of PARP inhibitors established the foundation for its clinical application in cancer treatment (76). PARP inhibitors also demonstrate immunoregulatory effects in preclinical studies. They can attenuate chronic inflammation and increase T cell infiltration (77).

Olaparib has been recently approved for the treatment of ovarian cancers with BRCA1 and BRCA2 mutations. Olaparib in combination with PD-L1 inhibitor, durvalumab, was recently investigated in a clinical trial for patients with gynecologic cancers. Among 26 enrolled women, a disease control rate of 83% was reported, with an acceptable safety profile (78). Several trials evaluating different PARP inhibitors in combination with ICIs are currently ongoing in various solid tumors, including NSCLC and breast cancer (Table 3). The combination of niraparib with pembrolizumab was studied in the phase 1/2 TOPACIO/Keynote-162 (NCT02657889) study, and the results from the recurrent ovarian cancer cohort were recently reported (79). Among the 60 evaluable patients, an ORR of 25% was seen in all platinum-resistant ovarian cancer patients with an ORR of 45% in those with BRCA mutations. No new safety concerns were identified.

**Table 3:** Clinical trials of immune checkpoint inhibitors in combination with poly (ADP-ribose) polymerase inhibitors or vascular endothelial growth factor targeting therapy.

Trial	Tumor type	Treatment regimen	Outcome
Lee et al. (78)	Gynecological cancers	Durvalumab + olaparib Durvalumab + cediranib	83% disease control rate in durvalumab + olaparib group 75% disease control rate in durvalumab plus cediranib group
NCT02734004	Breast, gastric, ovarian and SCLC	Durvalumab + olaparib	Ongoing
NCT02484404	NSCLC, SCLC, breast, ovarian, colorectal, prostate	Durvalumab + olaparib Durvalumab + cediranib Durvalumab + olaparib + cediranib	Ongoing
NCT02657889	Breast and ovarian	Pembrolizumab + niraparib	Ongoing
NCT02944396	NSCLC	Nivolumab + veliparib + platinum-based chemotherapy Veliparib + platinum-based chemotherapy	Ongoing
NCT02849496	Breast	Veliparib Atezolizumab Veliparib + atezolizumab	Ongoing
NCT02443324	Gastric, GEJ adenocarcinoma, NSCLC, transitional cell carcinoma of the urothelium, biliary tract	Pembrolizumab + ramucirumab	Ongoing
NCT02572687	GI or thoracic malignancies	Durvalumab + ramucirumab	Ongoing

Given the fact that DNA repair response not only regulates tumorigenesis but also plays role in antitumor immunity, it is imperative to fully understand the interplay between DNA repair inhibitors and ICIs to combine them in a safe and effective manner, since inhibition of DNA repair response can potentially decrease the immune response. Moreover, the intrinsic tumor genetic background and DNA repair response status [e.g., BRCA mutation vs. wild-type (WT)] have positive impact on the PARP inhibitor-induced antitumor immune response during immunotherapy. Furthermore, it is possible that different PARP inhibitors can modulate the antitumor immune response through different mechanisms, which could impact the treatment outcomes when combined with different ICIs.

### VEGF Inhibitors

Vascular endothelial growth factor stimulates angiogenesis, tissue remodeling, and fibrosis. Its immunosuppressive effects make VEGF a good target candidate to potentiate the antitumor immune response in combination with ICIs (80, 81). Studies from our group have shown that in patients with stage IV melanoma the baseline Treg concentration positively correlates with baseline VEGF level, which associates with poor clinical outcomes (82). Upregulated VEGF level and chronically Th-2-mediated immune status are observed in patients with metastatic melanoma (83). Animal models have shown that anti-VEGF antibody can increase T cell tumor infiltration with enhanced antitumor response (84). Decreased Treg proliferation and MDSC population are associated with bevacizumab treatment in CRC (85). The hypoxic conditions resulting from anti-VEGF treatment also can upregulate PD-L1 expression.

Tremelimumab in combination with sunitinib has been evaluated in patients with metastatic melanoma. Unfortunately, unexpected dose-limiting renal toxicity was observed (86). High dose of tremelimumab (6 mg/kg) used in this study could contribute to the adverse effects. Bevacizumab was also investigated in combination with ipilimumab in melanoma patients (87). A disease control rate of 67.4% was observed with combination therapy, with increased CD8^+^ lymphocyte infiltration resulting from more effective lymphocytic trafficking. Multiple clinical trials are currently ongoing investigating the combination of bevacizumab and pembrolizumab in patients with ovarian cancer (NCT02853318) and solid tumor brain metastases (NCT02681549). Other agents targeting VEGF pathway, such as ramucirumab, are also being evaluated in combination regimens (Table 3). Lenvatinib, a tyrosine kinase inhibitor, is being studied in combination with pembrolizumab in a phase 1/2 trial (NCT03006926). However, the dynamic immunologic effects of these combinations remain to be elucidated. In a recent report, 10 patients with metastatic renal cell carcinoma were treated with bevacizumab plus atezolizumab after bevacizumab run-in period (88). A partial disease response was observed in four patients, and median time to response was 4.2 months. Following combination therapy, increased intratumoral CD8^+^ T cells, Th1, and T effector markers are found. Interestingly, increases in intratumoral chemokine, CX3CL1, and peripheral CX3CR1^+^ (CX3CL1 receptor) CD8^+^ T cells are observed after combination therapy, similar to the observation in patients received CIT combination (53). Clinical trials are ongoing to evaluate this combination in untreated metastatic renal cell carcinoma (IMmotion151 study) (89) and untreated locally advanced or metastatic hepatocellular carcinoma (IMbrave150) (NCT03434379). Further investigation will be critical to design safe and efficacious combinations and to address biomarker selection.

## Combination of Immunotherapy with Radiotherapy (RT)

Radiotherapy remains to be the backbone modality in the treatment for different types of cancer, either given alone or in combination with chemotherapy. It induces single- and double-strand DNA breaks, triggering multiple signaling pathways including DNA damage responses and activation of cell cycle checkpoints. The RT-induced cell death can further initiate systemic antitumor responses through various immune cell subsets (90). The participation of immune cells is indispensable for the clinical benefit of RT; in turn, RT can also modulate the antitumor immunity. It has been reported that localized tumor radiation can result in distant systemic tumor control in the unirradiated area. This clinical phenomenon, known as the abscopal effect, is a result of RT-induced immune modulations (91). The potential synergistic antitumor activities of RT in combination with ICIs have attracted increased research efforts in this new era of cancer immunotherapy (92).

Increased infiltration of macrophages and monocytes has been observed post-irradiation in multiple humana cancer xenograft models (squamous cell carcinoma, breast, and lung carcinoma), and depletion of the TAMs with antibody to CD11b or inhibitor of SDF-1a receptor CXCR4 (AMD3100) provided further tumor control when combined with irradiation (93, 94). The activation of dendritic cells and their release of type I interferon after irradiation is also critical in CD8^+^ T cell activity and treatment efficacy in mouse colon carcinoma (MC38), lung carcinoma (LLC), and melanoma models (B16F10) (95, 96). In an animal model of Lewis lung carcinoma (LLC), when the local area was exposed to irradiation, distant tumor control was observed only in p53 WT (vs. in p53 null mice), suggesting that the abscopal effect was mediated through pathways downstream of p53 (97). Multiple studies have shown that cytotoxic CD8^+^ T cells are required for RT-induced tumor control in mouse breast cancer (4T1), melanoma (B16), lymphoma (EL4), and lung cancer (98, 99).

Although the myelosuppression after irradiation is thought to be immunosuppressive, RT can regulate T cell-mediated immune responses *via* various mechanisms (100). RT regulates key cell surface molecules for cytotoxic immune cell activation (NKG2D), and antigen presentation machinery (MHC class I expression with antigen peptides), therefore augments T cell tumor recognition and activation (101, 102). RT also enhances T cell priming for activation via activation of antigen-presenting cells (dendritic cells) and releasing of immunogenic antigens (95, 103). RT increases the release of chemokines (CXCL16, a chemokine that binds to CXCR6 on Th1 CD4 and effector CD8 T cells) by mouse breast cancer (4T1) cells, therefore enhances the infiltration of cytotoxic T cells (104). In this situation, blockade of CTLA-4 further promotes tumor regression. Irradiation also alters the immunosuppressive tumor microenvironment to a M1 phenotype, favoring accessibility for T cell infiltration (105). In addition, in melanoma and Kras-mutant lung cancer models, irradiation was found to upregulate PD-L1 expression in tumor microenvironment (106, 107). The synergistic relationship between RT and ICI has been further explored in preclinical models. In mice bearing poorly immunogenic breast carcinoma, treatment with CTLA-4 blockade in combination with RT (vs. CTLA-4 blockade alone) resulted in decreased tumor growth and metastasis with improved survival (108). RT in combination with PD-1 blockade also induced improved and durable tumor control in NSCLC mouse models (107). In melanoma and renal cell carcinoma animal models, PD-1 knockout (KO) mice demonstrated higher survival after stereotactic ablative radiotherapy (SABR) compared with their PD-1 WT little mates. The addition of PD-1 blocking antibody to SABR led to the improved antitumor response and survival in PD-1 WT mice. In addition, treatment with SABR and anti-PD1 antibody combination induced significant reduction of non-irradiate tumor. The increased frequency of CD11a^high^CD8^+^ tumor-reactive T cells and their enhanced functions were associated with the antitumor response in PD-1 KO mice, suggesting the translation potential of combining RT and PD-1 blockade (109).

Over the recent years, the clinical efficacy of RT in combination with ICIs have been studied in multiple clinical trials were designed to investigate. However, data supporting the routine application of this combination are still limited. The RT and ipilimumab combination demonstrated acceptable toxicity profile in patients with metastatic melanoma, yet failed to provide survival benefit (110). Ipilimumab in combination with radiation was evaluated in a single-arm phase 2 study in melanoma patients with unresectable brain metastases (111). Fifty-eight patients were enrolled in this study; with 1-year OS of 31.8% (95% CI [18.8–44.8%]) that is higher than the historical reported results, without unexpected adverse events.

In a phase 1 KEYNOTE-001 study, 97 patients with advanced NSCLC were enrolled (112). Longer PFS and OS, without higher incidence of grade 3 or above pulmonary toxicity, were seen in patients who underwent RT prior to anti-PD1 therapy compared with those who did not receive RT, supporting the safety and potential synergistic activity of the RT-ICI combination. The phase 3 randomized PACIFIC trial investigated the role of subsequent durvalumab therapy in stage III NSCLC patients after definitive chemoradiation (NCT02125461) (113). A total of 713 patients with locally advanced unresectable NSCLC without disease progression after definitive chemoradiation were randomly assigned in 2:1 to receive durvalumab or placebo irrespective of PD-L1 status. Median progression survival is 16.8 months in durvalumab group vs. 5.6 months in placebo group (16.8 months vs.), with 18-month PFS rate of 44.2 vs. 27.0%, respectively (113). Results from this study led to the FDA approval of durvalumab for the treatment of stage III NSCLC regardless of PD-L1 expression levels. The RT and immunotherapy combination is currently being evaluated in other tumor types, such as metastatic GI malignancies (NCT02830594) and metastatic breast cancer (NCT02730130) (Table 4).

**Table 4:** Clinical trials of immune checkpoint inhibitors in combination with radiotherapy (RT).

Trial	Tumor type	Regimens	Outcomes
Shaverdian et al. (112)	NSCLC (KEYNOTE-001)	Compared patients on pembrolizumab with previous RT to those who did not receive previous RT	Previous treatment with RT results in longer PFS and OS, with an acceptable safety profile
Antonia et al. (113)	NSCLC	Definitive ChemoRT → durvalumab Definitive ChemoRT → placebo	Progression-free survival (PFS): 16.8 months 18 months PFS: 44.2% PFS: 5.6 months 18 months PFS: 27.0%
Levy et al. (114)	Inoperable or metastatic cancers	Concurrent durvalumab + RT	Concurrent palliative RT with the anti-PD-L1 durvalumab was well tolerated
Tang et al. (115)	Metastatic solid tumor	Ipilimumab + stereotactic ablative radiotherapy (SABR)	Combining SABR and ipilimumab was safe with signs of efficacy, peripheral T-cell markers may predict clinical benefit, and systemic immune activation was greater after liver irradiation
Hiniker et al. (116)	Metastatic melanoma	Palliative RT + ipilimumab	Combination therapy was well tolerated without unexpected toxicities. Eleven patients (50.0%) experienced clinical benefit from therapy, including complete and partial responses
NCT03050554	NSCLC	Stereotactic body radiation therapy (SBRT) in combination with Avelumab	Ongoing Tolerability, RFS
NCT02658097	NSCLC	Single fraction nonablative radiation in combination with pembrolizumab	Ongoing RR and best OS
NCT03458455	Brain tumor	Stereotactic radiosurgery plus ipilimumab, nivolumab, or pembrolizumab	Ongoing Treatment response at 18 months
NCT03115801	Metastatic renal cell carcinoma and urothelial carcinoma	Nivolumab/atezolizumab Nivolumab/atezolizumab plus RT	Ongoing Best overall response rate, PFS, toxicities, OS
NCT03176173	NSCLC	Radical-dose image guided radiation therapy daily for up to 10 days (within 2 weeks) while undergoing standard of care immunotherapy Patients who decline to undergo radiation therapy receive standard of care immunotherapy	Ongoing PFS

Despite the encouraging finding from the PACIFIC trial, future research is urgently needed to define the immunoregulatory mechanisms that cross talk between RT, chemo, and immunotherapy, to design optimized combination strategies. Similar to other combination strategies discussed, the optimum sequencing of the RT and ICI combination has not been elucidated in available studies. In the KEYNOTE-001 study, irradiation prior to pembrolizumab therapy provided improved PFS and OS (112), while in another clinical report palliative RT-induced global disease control in a PD-1 antibody resistant patient (117). In a melanoma mouse model, the addition of adjuvant chemotherapy after ablative RT abrogated the RT-induced CD8^+^ T cell activation and tumor control, while the addition of immunotherapy can enhance the tumor response (98), suggesting that the treatment modalities and the sequence of their combination need to be carefully investigated to achieve clinical success. In addition, research is needed to identify biomarkers with both predictive and prognostic values in RT-immunotherapy combination.

## Conclusion

Modern cancer immunotherapies exert their tumor-killing activities through enhancing antitumor immunity while suppressing the tumor-promoting immune process. However, since ICIs do not provide clinical benefits in the majority of cancer patients, it is crucial to design rational and efficacious synergic therapeutic approaches to increase clinical responses to ICI. The combination of chemotherapy, targeted therapy, and RT with ICIs has gained increased attentions from clinicians and researchers over the recent years, given their immunomodulatory effects and potential synergistic antitumor activities. Despite the encouraging clinical results from various clinical studies, further investigations are warranted to elucidate the exact molecular and cellular mechanisms driving these clinical responses. More importantly, the optimal regimens, dose, timing, and schedule of the combination therapy for differently types of tumors are yet to be identified. Molecular interplay between different therapeutic modalities will need to be further investigated given the unlimited possibilities of combining currently available cancer treatments.

## Author Contributions

YY and HD conceived the outline of the manuscript. YY, HD, AK, HF, and SM wrote and edited the manuscript. SP and RD edited the manuscript.

## Conflict of Interest Statement

The authors declare that the research was conducted in the absence of any commercial or financial relationships that could be construed as a potential conflict of interest.
